# Characterization and Expression Profiling of *Camellia sinensis* Cinnamate 4-hydroxylase Genes in Phenylpropanoid Pathways

**DOI:** 10.3390/genes8080193

**Published:** 2017-08-01

**Authors:** Jinxin Xia, Yajun Liu, Shengbo Yao, Ming Li, Mengqing Zhu, Keyi Huang, Liping Gao, Tao Xia

**Affiliations:** 1 School of Life Science, Anhui Agricultural University, Hefei 230036, Anhui, China; JinxinXia@ahau.edu.cn (J.X.); liuyajun1228@163.com (Y.L.); liming4802@163.com (M.L.); mengqing1234567@163.com (M.Z.); huluobuhashiqi@163.com (K.H.); 2 State Key Laboratory of Tea Plant Biology and Utilization, Anhui Agricultural University, 130 West Changjiang Rd, Hefei 230036, Anhui, China; yaoandtea@163.com

**Keywords:** *Camellia sinensis*, phenylpropanoid, cinnamate 4-hydroxylase, expression pattern, abiotic stress

## Abstract

Cinnamate 4-hydroxylase (C4H), a cytochrome P450-dependent monooxygenase, participates in the synthesis of numerous polyphenoid compounds, such as flavonoids and lignins. However, the *C4H* gene number and function in tea plants are not clear. We screened all available transcriptome and genome databases of tea plants and three *C4H* genes were identified and named *CsC4Ha*, *CsC4Hb*, and *CsC4Hc*, respectively. Both *CsC4Ha* and *CsC4Hb* have 1518-bp open reading frames that encode 505-amino acid proteins. *CsC4Hc* has a 1635-bp open reading frame that encodes a 544-amino acid protein. Enzymatic analysis of recombinant proteins expressed in yeast showed that the three enzymes catalyzed the formation of *p*-coumaric acid (4-hydroxy *trans*-cinnamic acid) from *trans*-cinnamic acid. Quantitative real-time PCR (qRT-PCR) analysis showed that *CsC4Ha* was highly expressed in the 4th leaf, *CsC4Hb* was highly expressed in tender leaves, while *CsC4Hc* was highly expressed in the young stems. The three *CsC4Hs* were induced with varying degrees by abiotic stress treatments. These results suggest they may have different subcellular localization and different physiological functions.

## 1. Introduction

Tea, one of the most popular non-alcoholic beverages in the world, is rich in polyphenoid compounds derived from phenylpropanoid pathways, e.g., catechins (flavan-3-ols), flavonols, and their derivatives [1]. These compounds are closely related to the flavor of tea [2] and benefit human health through their anti-retroviral, anti-hypertensive, anti-inflammatory, anti-aging, and insulin-sensitizing activities. In addition, these compounds inhibit low-density lipoprotein (LDL) oxidation and reduce the risk of a wide range of chronic diseases, including cardiovascular disease, cancer, and osteoporosis [3].

The core reactions in phenylpropanoid biosynthesis that produce important secondary metabolites participating in plant development and defense responses [4] involve three enzymes, phenylalanine ammonia-lyase (PAL; EC 4.3.1.5), cinnamate 4-hydroxylase (C4H, EC 1.14.13.11), and 4-coumarate: coenzyme A ligase (4CL; EC 6.2.1.12). C4H, a member of the cytochrome P450 family [5], catalyzes the formation of *p*-coumaric acid (4-hydroxy *trans*-cinnamic acid) from *trans*-cinnamic acid.

**Figure 1:**
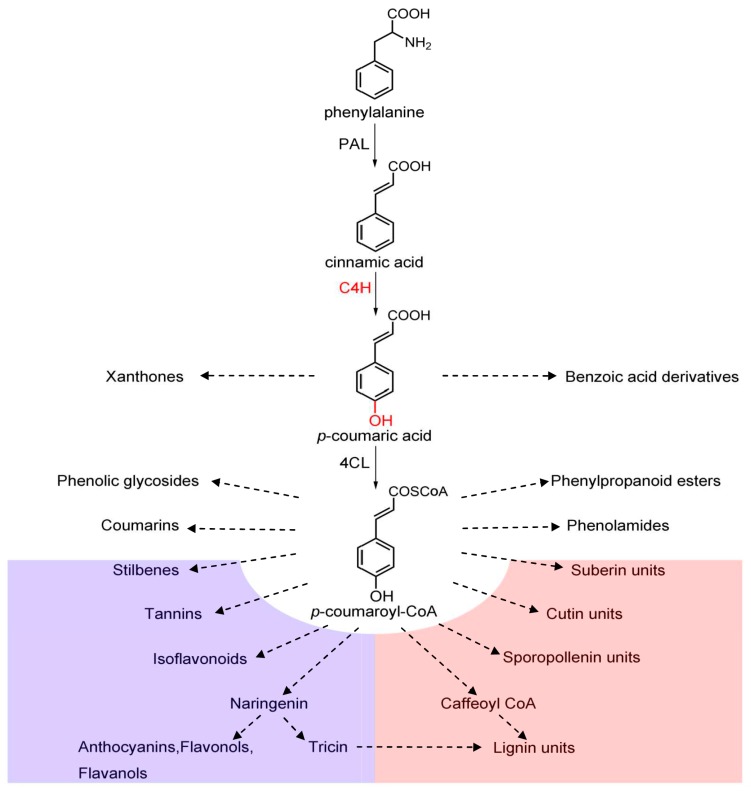
Schematic view of some branches of the phenylpropanoid pathway. PAL: phenylalanine ammonia-lyase; C4H: cinnamate 4-hydroxylase; 4CL: 4-coumarate: coenzyme A ligase.

As the upstream enzyme, C4H forms 4-coumarate (or *para*-coumarate), which is used to yield coenzyme A (CoA)-thioester by 4CL. 4-Coumaroyl-CoA is the precursor for many phenylpropanoid compounds including flavonoids and lignin[6] (Figure 1). Lignin plays an important role in plant defense mechanisms against pathogens [7,8]. Flavonoids, which originate from the flavonoid synthetic pathway located downstream of the phenylpropanoid pathway, include flavanols, anthocyanins, proanthocyanidins, flavonols, and flavones. Flavonoids protect plants from different biotic and abiotic stresses, act as unique ultraviolet (UV)-filters, and function as signal molecules, allelopathic compounds, phytoalexins, detoxifying agents, and antimicrobial defense compounds [9].

C4H, a member of the cytochrome P450 CYP73A group, has been most extensively studied among plant P450s [10,11,12,13,14]. The number of *C4H* gene families varies considerably between different plants. *Arabidopsis*, *Parthenocissus henryana*, parsley, *Scutellaria baicalensis*, and Korean black raspberry are thought to contain only one gene for C4H [13,15,16]. On the contrary, in *Leucaena leucocephala*, *Camptotheca acuminata*, and *Brassica napus*, C4H is encoded by a small gene family [5,17,18].

In the currently online available database of transcriptomes and genomes of tea plants, three *CsC4H* transcripts have been screened, but their functions in tea plants remain unclear. For example, it remains to be determined whether their coding candidate *Cs*C4H enzymes have enzymatic activities or whether these genes participate in the response of tea plants to biotic or abiotic stresses.

In this study, we cloned the three genes, *CsC4Ha*, *CsC4Hb*, and *CsC4Hc*, verified their enzymatic functions using yeast recombinant proteins, and analyzed their expression profiles in tea plants subjected various abiotic stresses.

## 2. Materials and Methods

### 2.1. Plant Materials

Samples of the tea plant *Camellia sinensis* cv. ‘Shucazao’ were obtained from the experimental tea garden of Anhui Agricultural University in Hefei, China. Leaves at six different developmental stages (bud, 1st leaf, 2nd leaf, 3rd leaf, 4th leaf, and mature leaf), and young stems and young roots were collected. The samples were immediately frozen in liquid nitrogen and stored at −80 °C.

With respect to the abiotic stresses, the approximately 10 cm long shoots were cultured in water for one day and then subjected to the treatments. The samples were treated under 100 mM abscisic acid (ABA) and 90 mM sucrose for 12 h, 20 mM salicylic acid (SA) treatment for 48 h, respectively. The control plants were cultivated in deionized water. For heat stress, the shoots were treated at 50 °C for 30 min with the controls treated at 20 °C.

The tender shoots were illuminated under ultraviolet radiation b (UVB) for 30 min, blue light (455–460 nm) for 48h, red light (655–660 nm) for 48 h, and in dark for 12 h, respectively. The control plants were treated under white light.

All samples were immediately frozen in liquid nitrogen, and total RNA was extracted as described below.

The yeast strain (*Saccharomyces cerevisiae* cv. WAT11) was kindly provided by Conagen Inc. (Bedford, MA, USA).

### 2.2. RNA and cDNAPreperation

Total RNA was extracted from the tea plants using RNAiso Mate and RNAiso Plus (Takara, Dalian, China) according to the manufacturer’s instructions. The quality of the RNA was checked using gel electrophoresis, and total RNA was quantified using NanoVue plus (GE Healthcare, Waukesha, WI, USA). The different cDNAs were reverse transcribed using a PrimeScript RT Reagent Kit (Takara), following the manufacturer’s protocol.

### 2.3. Cloning of CsC4Ha, CsC4Hb, and CsC4Hc

The databases accessed in the National Center for Biotechnology Information (NCBI) for screening the candidate genes include nine transcriptome databases, such as ‘Fudingdabaicha’ (TSA: GAAC01000001-GAAC01052919), ‘Longjing43’ (TSA: GBBZ01000001-GBBZ01049010, HP701085-HP777243), ‘Xinyangdaye’ (TSA: GEFQ01000001-GEFQ01129526), ‘Zhongcha302’ (TSA: GBKQ01000001-GBKQ01063798), ‘Shuchazao’ (from our laboratory), ‘Huangkui’ (from our laboratory), ‘Ziya’ (from our laboratory), ‘Teenali’ (TSA: GBRC01000001-GBRC01033639), ‘Hongye2’ (TSA: KA279444-KA304315), and a genome database (www.plantkingdomgdb.com/tea_tree/).

The *CsC4H* mRNAs from the transcriptome database were subjected to standard polymerase chain reaction (PCR) reactions, with the primers designed according to the cDNA sequence (synthesized by the Beijing Genomics Institute, Beijing, China; Table 1). The cDNA strands for end-to-end PCR were synthesized using Phusion High-Fidelity DNA Polymerase (Thermo Scientific, Vilnius, Lithuania). PCR products were gel purified using an agarose gel extraction kit (Takara), ligated into a pEASY-Blunt Simple T vector (TransGen Biotech, Beijing, China), and transformed into *Escherichia coli* DH5α competent cells for sequencing. The results were assembled using DNAMAN V6 software (Lynnon Corporation, San Ramon, CA, USA). Briefly, end-to-end PCR was performed under the following conditions: 98 °C for 30 s, 30 cycles at 98 °C for 10 s, 56 °C for 30 s, 72 °C for 90 s, and a final extension at 72 °C for 10 min.

### 2.4. Quantitative Real-Time PCR 

All primers were blasted against the NCBI database (United States National Library of Medicine, Bethesda, MD, USA) to guarantee specificity. The values were normalized against the expression levels of the housekeeping gene glyceraldehyde-3-phosphate dehydrogenase (*GAPDH*) from the tea plant [19]. The first-strand cDNA samples for quantitative real-time PCR (qRT-PCR) were synthesized using the PrimeScript RT reagent Kit (Takara). The PCR mixture contained cDNA template (approximately 0.01 μg/μL), 10 μL SYBR Green PCR Master Mix (Takara), and 200 nmol/L of each gene-specific primer in a final volume of 20 μL.

Real-time PCR was performed as suggested by Lei Zhao et al. [20]. Data were expressed as the mean value of three biological replicates, normalized against the expression levels of *GAPDH*. The relative expression was derived using the 2^−ΔΔCT^ method. ΔCT = CT_target_ − CT_internal standard_, −ΔΔCT = −(ΔCT_target_ − ΔCT_control_), where CT_target_ and CT_internal standard_ are the cycle threshold (CT) values for the target and housekeeping genes, respectively.

### 2.5. Heterologous Expression and Enzymatic Activity Analysis of Recombinant CsC4Hs

The PCR products of *CsC4Ha*, *CsC4Hb*, and *CsC4Hc*, obtained using end-to-end PCR, were gel purified and ligated into pENTR/TEV/D-TOPO vectors using Top cloning (pENTR /D-TOPO Cloning Kits, Invitrogen, Carlsbad, CA, USA). Then, the entry vectors pENTR-*Cs*C4Ha, pENTR-*Cs*C4H,b and pENTR-*Cs*C4Hc were cloned into the destination vector pYES-DEST52 using the Gateway LR Clonase enzyme (Invitrogen, Carlsbad, CA, USA). The resulting pYES-DEST52-*Cs*C4Ha, pYES-DEST52-*Cs*C4Hb, and pYES-DEST52-*Cs*C4Hc were transformed into *S. cerevisiae* WAT11 using Frozen-EZ yeast Transformation II (Zymo Research, Irvine, CA, USA). Yeast cells were propagated at 28 °C for 12 h in 10-mL Synthetic Dropout-Ura Media (SD-U) liquid medium containing 20 g/L glucose, by inoculation of a single colony from an SD-U plate. The thalli collected were transferred into 10-mL SD-U medium containing 20 g/L galactose and were grown at 28 °C for 5 h. The substrate *t*-cinnamate was added to the yeast culture to a final concentration of 0.2 mM and incubated at 28 °C for 1 h. The reactions were terminated by sonication for 15 min and the addition of methanol. Microzymes from each reaction were extracted using the same volume of methanol after high-speed centrifugation (1300 rpm, 160× *g*, 10 min) and 0.22-μm membrane filtration for high-performance liquid chromatogram (HPLC) analysis at 275 and 309 nm.

HPLC: After removal of the denatured proteins by centrifugation, the formation of *p*-coumaric acid was analyzed using a HPLC equipped with an Altima C18 analytical column (250 mm × 4.6 mm, 5 μm) (Agilent, Santa Clara, CA, USA) with a gradient elution of solvent B (CH_3_CN) and solvent A (1% acetic acid) at a flow rate of 1 mL/min at 35 °C over a 30-min period as follows: 0 min, 10% solvent B; 5 min, 15% solvent B; 15 min, 40% solvent B; 20 min, 60% solvent B; 25 min, 80% solvent B, and 30 min, 10% solvent B. A diode array detector (DAD) was used for monitoring purposes. All experiments were performed in duplicate.

### 2.6. Bioinformatics Analysis

Cinnamate 4-hydroxylase candidate genes were analyzed using online bioinformatics tools from NCBI and ExPASy (SIB Swiss Institute of Bioinformatics, Lausanne, Switzerland). Open reading frame (ORF) identification was performed using an online program (National Center for Biotechnology Information, U.S. National Library of Medicine, Bethesda, MD, USA) [21]. The amino acid sequence of the ORF was deduced and analyzed using the ProtParam tool (National Center for Biotechnology Information, U.S. National Library of Medicine) [22]. *Cs*C4Hs and other known C4H sequences retrieved from NCBI database (U.S. National Library of Medicine) were aligned with DNAMAN (Lynnon Corporation, San Ramon, CA, USA) [20]. Subsequently, a phylogenetic tree was constructed using the neighbor-joining (NJ) method with MEGA 5.0 software (Mega, Raynham, MA, USA). The reliability of the tree was measured using bootstrap analysis based on 1000 replicates.

## 3. Results

### 3.1. Screening, Analysis, and Cloning of CsC4H Candidate Genes

After careful analysis of the *CsC4H* sequences based on the nine transcriptome databases and one genome database, three *CsC4H* transcripts were screened out after removing redundancies. The accession numbers of the three *CsC4H* genes in GenBank are KY615675 (*Cs*C4Ha), KY615676 (*Cs*C4Hb), and KY61567 (*Cs*C4Hc). *CsC4Ha*, *CsC4Hb*, and *CsC4Hc* were isolated using PCR, with cDNA from *Camellia sinensis* leaves as a template using specific primers (Table 1). Both *CsC4Ha* and *CsC4Hb* have a 1518-bp open reading frames that code for a 505-amino acid protein. They have a predicted molecular mass of 58.15 kDa and 58.00 kDa and a predicted isoelectric point (pI) of 9.29 and 9.26, respectively. *CsC4Hc* has a 1635-bp open reading frame that codes for a 544-amino acid protein, with a predicted molecular mass of 62.95 kDa and a pI of 8.68 (Figure S1).

### 3.2. Bioinformatics Analysis

DNAMAN software (Lynnon Corporation, San Ramon, CA, USA) analysis shows that the amino acid sequences of *Cs*C4Ha share 93.07% identity with that of *Cs*C4Hb. The amino acid sequences of *Cs*C4Ha and *Cs*C4Hb share only 59.93% and 58.82% identity with *Cs*C4Hc, respectively.

**Figure 2:**
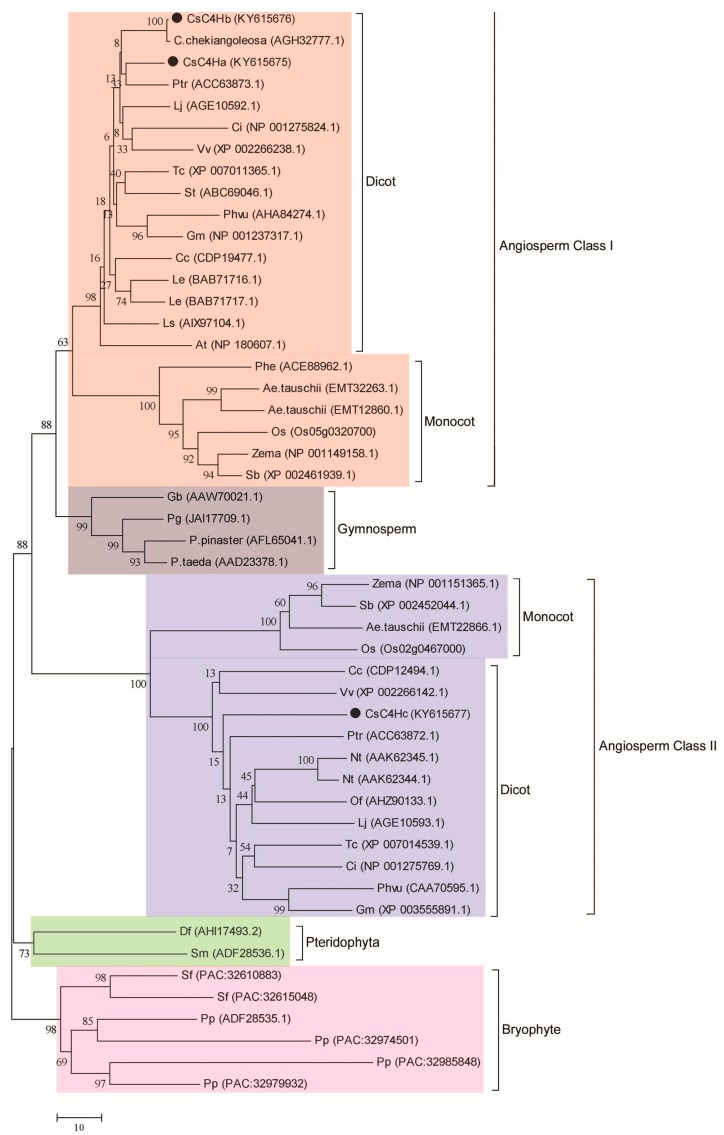
Phylogenetic tree of *Cs*C4Hs and C4Hs from other plants. The bars represent the evolutionary distance. *Cs*C4Hs are indicated by a circle (●). The following sequences were analyzed: *Camellia sinensis* C4Hb (KY615676), *Camellia chekiangoleosa* (AGH32777.1), *C. sinensis* C4Ha (KY615675), *Populus trichocarpa* (ACC63873.1), *Citrus sinensis* (NP_001275824.1), *Vitis vinifera* (XP_002266238.1), *Lonicera japonica* (AGE10592.1), *Theobroma cacao* (XP_007011365.1), *Solanum tuberosum* (ABC69046.1), *Phaseolus vulgaris* (AHA84274.1), *Glycine max* (NP_001237317.1), *Coffea canephora* (CDP19477.1), *Lithospermum erythrorhizon* (BAB71716.1), *L. erythrorhizon* (BAB71717.1), *Lactuca sativa* (AIX97104.1), *Arabidopsis thaliana* (NP_180607.1), *Phyllostachys edulis* (ACE88962.1), *Aegilops tauschii* (EMT32263.1), *A. tauschii* (EMT12860.1), *Oryza sativa* (Os05g0320700), *Zea mays* (NP_001149158.1), *Sorghum bicolor* (XP_002461939.1), *Ginkgo biloba* (AAW70021.1), *Picea glauca* (JAI17709.1), *Pinus pinaster* (AFL65041.1), *Pinus taeda* (AAD23378.1), *Z. mays* (NP_001151365.1), *S. bicolor* (XP_002452044.1), *A. tauschii* (EMT22866.1), *O. sativa* (Os02g0467000), *C. canephora* (CDP12494.1), *V. vinifera* (XP_002266142.1), *C. sinensis* C4Hc (KY615677), *P. trichocarpa* (ACC63872.1), *Nicotiana tabacum* (AAK62345.1), *N. tabacum* (AAK62344.1), *Osmanthus fragrans* (AHZ90133.1), *Lonicera japonica* (AGE10593.1), *T. cacao* (XP_007014539.1), *C.s sinensis* (NP_001275769.1), *P. vulgaris* (CAA70595.1), *G. max* (XP_003555891.1), *D. fragrans* (AHI17493.2), *Selaginella moellendorffii* (ADF28536.1), *Sphagnum fallar* (PAC:32610883), *S.* *fallar* (PAC:32615048), *Physcomitrella patens* (ADF28535.1), *P. patens* (PAC:32974501), *P. patens* (PAC:32985848), and *P. patens* (PAC:32979932).

To investigate the evolutionary relationship among *Cs*C4Hs and C4Hs from other plant species, a phylogenetic tree was constructed using a neighbor-joining method. As shown in Figure 2, the phylogenetic tree was divided into five main groups: including Angiosperm Class I group (dicot and monocot), Angiosperm Class II group (dicot and monocot), Gymnosperm group, Bryophyte group, and Pteridophyte group. *Cs*C4Ha and *Cs*C4Hb belong to Angiosperm Class I and *Cs*C4Hc belongs to Angiosperm Class II.

**Figure 3:**
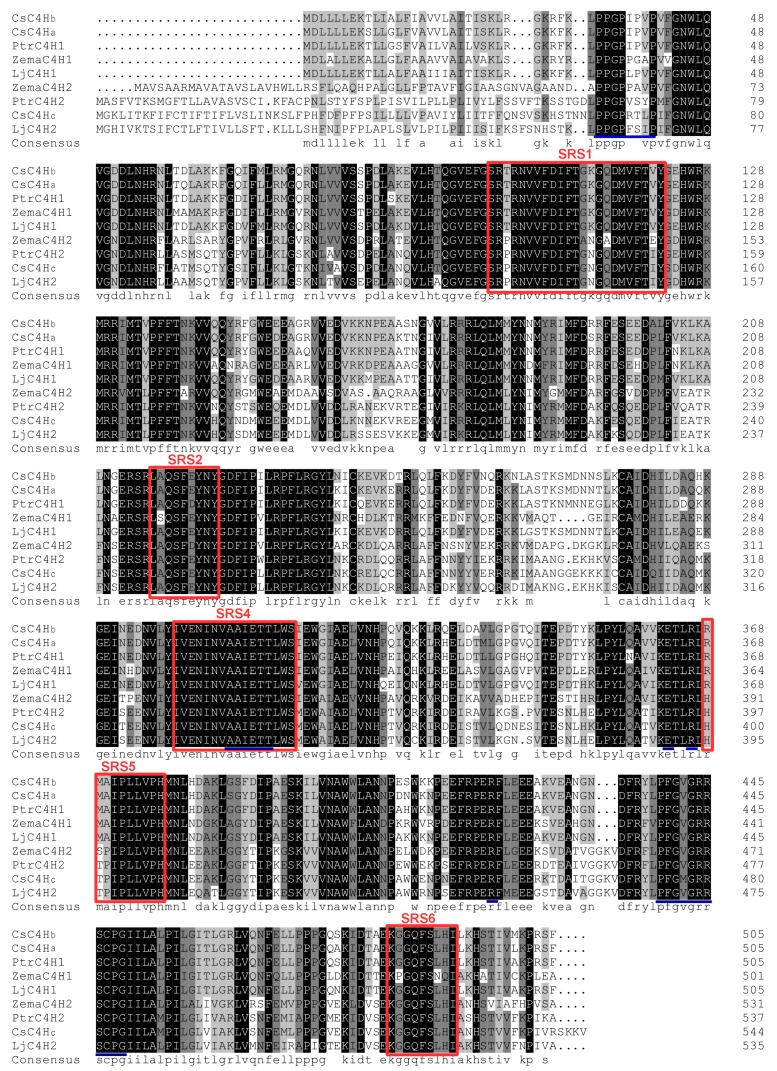
Multiple sequence alignment of *Cs*C4Hs with C4Hs from other plants. The following sequences are analyzed: *C. sinensis* C4Hb (*Cs*C4Hb, KY615676), *C. sinensis* C4Ha (*Cs*C4Ha, KY615675), *P. trichocarpa* C4H1 (*Ptr*C4H1, ACC63873.1), *Z. mays* C4H1 (*Zm*C4H1, NP_001149158.1), *L. japonica* C4H1 (*Lj*C4H1, AGE10592.1), *Z. mays* C4H2 (*Zm*C4H2, NP_001151365.1), *P. trichocarpa* C4H2 (*Ptr*C4H2, ACC63872.1), *C. sinensis* C4Hc (*Cs*C4Hc, KY615677), and *L. japonica* C4H2 (*Lj*C4H2, AGE10593.1). Completely identical residues are reverse-displayed, while residues with dark gray, light gray, and white backgrounds are conserved, weakly similar, and non-similar residues, respectively. Underlined regions indicate P450-featured motifs, i.e., the hinge region, the T-containing binding pocket motif, the ERR triad and the Haem-domain, while boxes represent the five P450 substrate recognition sites (SRS) regions.

Figure 3 shows the amino acid sequence alignment of three *C. sinensis* C4Hs with known functional C4Hs in other plants. There are five SRS (P450 substrate recognition sites) regions in these sequences, in addition to a heme-binding domain (P_474_FGXGRRSCPG_484_) and a hinge region (P_64_PGPXXXP_72_). The Angiosperm Class I proteins, including *Cs*C4Ha and *Cs*C4Hb, are conserved at the N-terminal and have a 21-amino acid N-terminal hydrophobic domain which is flanked by an acidic residue (Asp) and several basic residues [23]. This region is predicted to be a signal-anchor sequence and to determine the correct orientation of P450s in the Endoplasmic Reticulum (ER) [24]. However, the Angiosperm Class II, including the *Cs*C4Hc sequence, is not conserved in this region, which may result in different subcellular localization and different physiological functions.

### 3.3. Heterologous Expression in Yeast and Enzymatic Analysis of CsC4H Proteins

**Figure 4:**
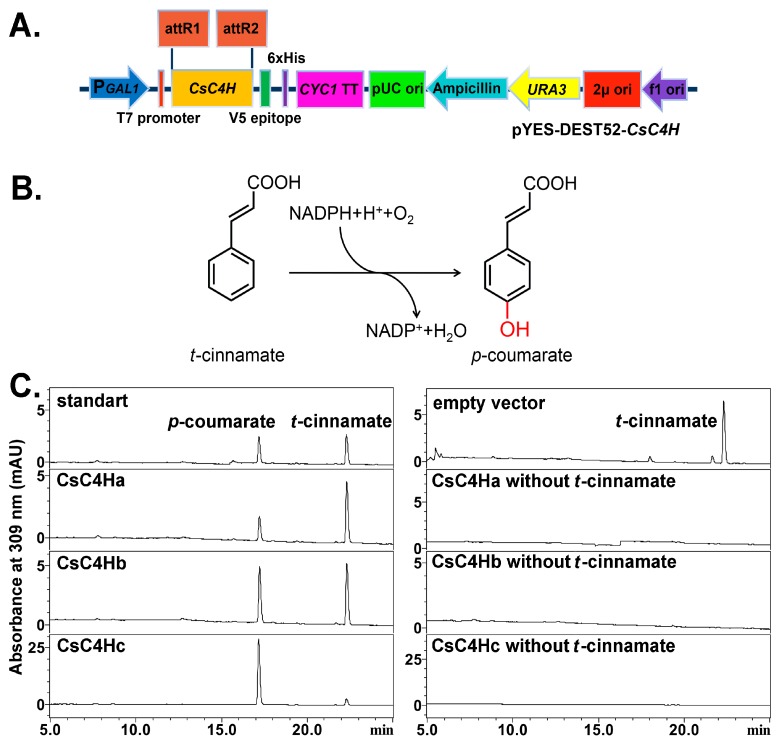
Heterologous expression in yeast and enzymatic activity analysis of *Cs*C4H proteins. (**A**) The expression vector of pYES-DEST52-*Cs*C4Hs; and (**B**) The hydroxylation of *t*-cinnamate to *p*-coumarate was catalyzed by *Cs*C4H. (**C**) The left panel shows the HPLC results of standard *t*-cinnamate and *p*-coumarate and the reaction products of WAT11 (pYES-DEST52-*Cs*C4Hs) using *t*-cinnamate as the substrate; the right panel shows the HPLC results of the control empty vector and WAT11 (pYES-DEST52-*Cs*C4Hs) without *t*-cinnamate as the substrate. Detection was performed at 309 nm.

We expressed *Cs*C4Hs recombinant proteins in a genetically modified *S. cerevisiae* strain, i.e., WAT11. PCR products of *CsC4Ha*, *CsC4Hb*, and *CsC4Hc* were ligated into the destination vector pYES-DEST52 (Figure 4A). The resulting pYES-DEST52-*Cs*C4Ha, pYES-DEST52-*Cs*C4Hb, and pYES-DEST52-*Cs*C4Hc were transformed into *S. cerevisiae* WAT11. The enzymatic catalytic identity of the three proteins was verified by enzyme assays using *trans*-cinnamic acid as a substrate, which was added to the yeast culture. The product *p*-coumarate was detected by HPLC analysis using an empty vector, and pYES-DEST52-*Cs*C4Hs without a substrate as controls, respectively (Figure 4C). The peak area of the enzymatic product indicates that three recombinant *Cs*C4Hs are able to convert *t*-cinnamate to yield *p-*coumarate when expressed in yeast.

### 3.4. Real-Time PCR Analysis of C4H Genes Expression in C. sinensis

**Figure 5:**
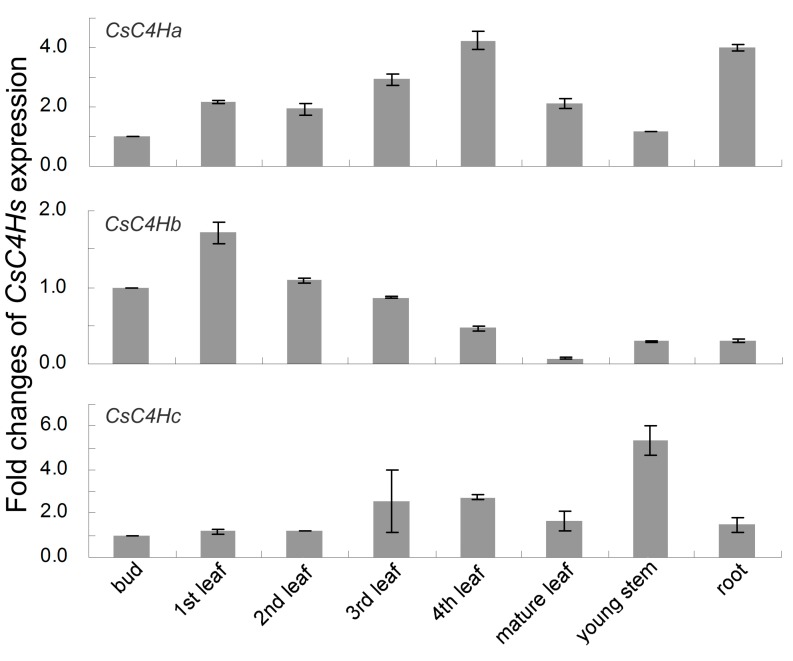
Expression pattern of *CsC4H* genes in various tissues based on quantitative real-time analysis. The normalized transcripts in the buds were set arbitrarily to one. The data represent the mean standard deviation from three independent measurements.

To analyze the expression patterns of the *CsC4H* genes in various tissues and at different developmental stages, quantitative real-time PCR was performed using gene-specific primers. The expression patterns of the three *CsC4H* genes are found to be distinct from each other (Figure 5) *CsC4Ha* is highly expressed in the 4th leaf and the roots. The expression level of *CsC4Hb* in tender leaves is significantly higher than that in old leaves, stems, and roots, which is consistent with the flavonoid accumulation pattern in tea plants [19]. Among the various tissues, *CsC4Hc* is mainly expressed in the young stems. In leaves at different developmental stages, *CsC4Hc* is relatively highly expressed in old leaves compared with tender leaves.

**Figure 6:**
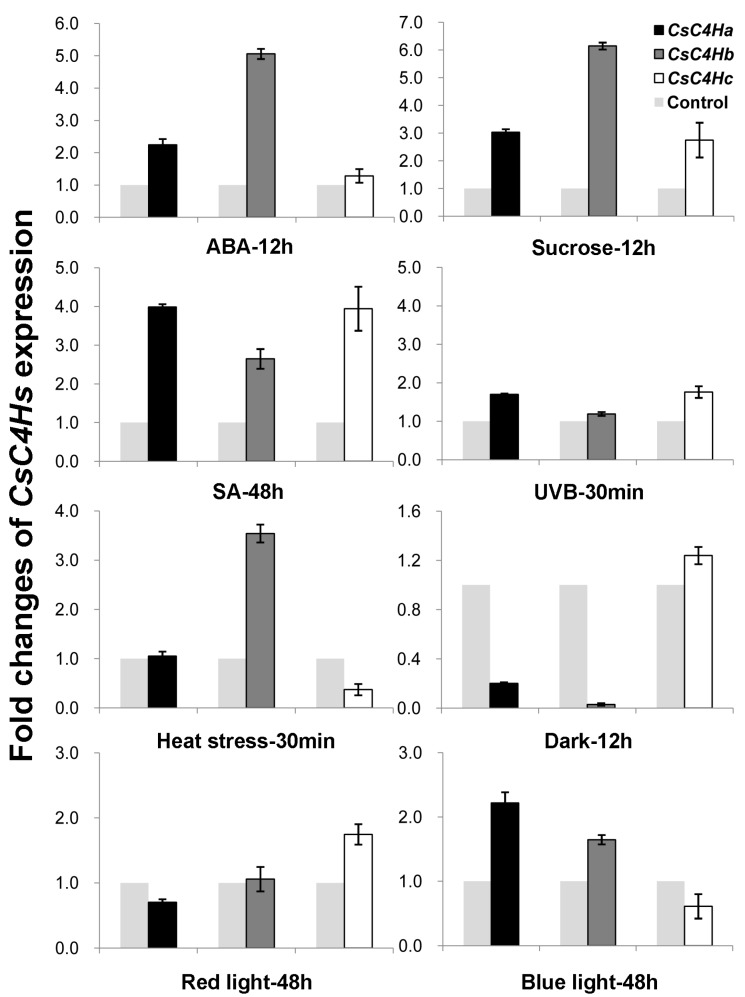
The transcript levels of *CsC4Hs* under different abiotic stresses. ABA: abscisic acid; SA: salicylic acid; UVB: ultraviolet radiation b.

Phenylpropanoid compounds can be induced by various biotic or abiotic stresses, such as high UV light intensity, wounding, and pathogen attack [25]. Therefore, we analyzed the inducible expression patterns of *CsC4H* genes in response to different abiotic stresses, including UVB, heat stress, ABA, sucrose, dark conditions, SA, red light, and blue light (Figure 6). The results show that the levels of the three genes are increased approximately 2–6-fold in the sucrose and SA treatments compared to the control. ABA and blue light significantly increase the expressions of *CsC4Ha* and *CsC4Hb*. By contrast, darkness treatment decreases the expressions of *CsC4Ha* and *CsC4Hb*. In addition, *CsC4Hb* is up-regulated under heat stress.

The *CsGAPDH* gene was used as an internal control. Induced expression analysis of *CsC4Hs* in tea leaves subjected to 100 mM ABA, 90 mM sucrose or dark treatment for 12 h. Induced expression analysis of *CsC4Hs* in tea leaves under 20 mM SA and in tea leaves treated with red (655–660 nm) or blue light (655–660 nm) for 48 h. Induced expression analysis of *CsC4Hs* in tea leaves exposed to UVB or heat stress for 30 min was conducted. The data represent the mean standart deviation (SD) from three independent measurements.

## 4. Discussion

Tea leaves of *C. sinensis* are an important non-alcoholic beverage resource [26]. The tea beverage is becoming increasingly popular worldwide because of its refreshing, mild stimulatory and medicinal properties [27]. Thus, research on the genes encoding crucial metabolic enzymes that are responsible for the biosynthesis of chemicals is essential and critical in understanding plant metabolic pathways. *C4H* is a key gene in the phenylpropanoid pathway; their function determines the downstream synthesis of flavonoid compounds and lignin.

*C4H* genes are known to exist as small gene families in various plants. For example, four homologous genes of *C4H* have been detected in *Populus tremuloides* and *P. kitakamiensis* [28]. In addition, two *C4H* genes have been detected in *Leucaena leucocephala* [5], and there are at least two *C4H* genes in *C. acuminata* and *Brassica napus* [17,18]. However, C4H is encoded by a single-copy gene in many species, such as *Arabidopsis*, parsley, *P. henryana*, and *S. baicalensis* [13,15,16,29]. To confirm the numbers of *CsC4H* members, we carefully screened all available transcriptome and genome databases, and three *CsC4H* transcripts were screened out after redundancies were removed. Three *C4H* transcripts were cloned from*C. sinensis*, which indicates there are at least three *C4H* genes in the tea genome. The addition of a substrate to a yeast culture experiment showed that the three recombinant C4H proteins had enzymatic activities that resulted in the formation of 4-coumarate (or *para*-coumarate). Quantitative expression analysis indicated different expression patterns of the *CsC4Hs* in various tissues and under abiotic stresses. The tissue- and induction-specific expressions of *CsC4Hs* indicated their different functions in vivo. 

According to the phylogenetic tree, the angiosperms were classified into two groups (Class I and Class II dicot and monocot). *Cs*C4Ha and *Cs*C4Hb belong to Class I dicot and monocot, whereas *Cs*C4Hc belongs to Class II dicot and monocot. This branch of the phylogenetic tree indicates that gene duplication prior to the divergence of monocots and dicots led to the divergent isoforms in angiosperms [23]. However, some dicots have lost their Class II protein, and the complete sequence of the *Arabidopsis* genome revealed no Class II gene [15].

Different functions of C4Hs in Class I and Class II have been reported. Eucalyptus Class II *C4H* is primarily involved in stress responses, as well as in wood lignin biosynthesis, and Class I *C4H* is constitutively expressed in any tissue that requires phenylpropanoid metabolites [30]. There are three *C4H* gene models in the *P. trichocarpa* genome. Transcripts of *PtrC4H1* and *PtrC4H2* (belonging to Class I) are abundant in differentiating xylem, suggesting that both are important in monolignol biosynthesis. Transcripts of *PtrC4H3* (belonging to Class II), not previously characterized, have been shown to have low or no expression in all examined tissues [31]. *C4H* genes from *P. tremuloides* and *P. trichocarpa* are differentially expressed in tissues, and individual isoforms have been shown to play specific physiological roles in development [32]. In this work, the expression patterns of *CsC4Hb* belonged to Class I in different tissues is consistent with the flavonoid accumulation pattern [19], indicating that *CsC4Hb* are involved in flavonoid biosynthesis in tea plants.

Phenylpropanoid compounds can be induced by various biotic and abiotic stresses [25]. *C4H* is induced by light; UVB, such as in *Arabidopsis*, *Dryopteris fragrans*, and *Salvia miltiorrhiza* [33,34,35]; wounding [36]; NaCl [37]; cold; H_2_O_2_; ABA; SA, such as in *Carthamus tinctorius* and kenaf [38,39]; and pathogen attack, for example, in cucumber and melon plants, *C4H* is up-regulated by viruses [40]; drought [41]; and elicitors [42]. Our work showed that the Cs*C4Ha* in Class I and Cs*C4Hc* in Class II were obviously induced by SA. This suggests that these two genes are involved in the defense of tea plants.

## 5. Conclusions

We cloned three *CsC4H* transcripts, and the enzymatic activity of these proteins was characterized in vitro. The amino acid sequence alignment of *Cs*C4H proteins and expression patterns of *CsC4H* genes in leaves at different developmental stages and abiotic stress treatments suggest they may have different subcellular localization and different physiological functions. The future work is under way.

## Figures and Tables

**Table 1:** Primers used in the study.

Gene	Primer	Primer Sequence	Use
*CsC4Ha*	*CsC4Ha*- F	5′-ATGGATCTTCTCCTCCTAGAGAAG-3′	Cloning
	*CsC4Ha*- R	5′-TCAGAACGATCTTGGTTTCAGAAC-3′	Cloning
	qPCR-F	5′-GCTCTCTGGCTATGACATCCCT-3′	qRT-PCR
	qPCR-R	5′-TCCTCCTTCCGACACCAAACG-3′	qRT-PCR
*CsC4Hb*	*CsC4Hb*- F	5′-ATGGATCTTCTTCTCCTAGAG-3′	Cloning
	*CsC4Hb*- R	5′-TTAAAATGATCTTGGTTTCATC-3′	Cloning
	qPCR-F	5′-GCTCGGCAGCTATGACATCC-3′	qRT-PCR
	qPCR-R	5′-CTCCTCCTACCAACACCGAATG-3′	qRT-PCR
*CsC4Hc*	*CsC4Hc*- F	5′-ATGGGCAAACTTATTACAAAATTTAT-3′	Cloning
	*CsC4Hc*- R	5′-TTAAACTTTTTTTGAACGAACAATTG-3′	Cloning
	qPCR-F	5′-GCGATGAAATCTCAACCGTCC-3′	qRT-PCR
	qPCR-R	5′-TGACCACAACCTTTGACTCCTTAG-3′	qRT-PCR

qRT-PCR: quantitative real-time PCR.

## Supplementary Materials

The following are available online at www.mdpi.com/2073-4425/8/8/193/s1. Figure S1: Gel electrophoresis of PCR to clone *CsC4Hs.*
