# Supplementary figures and images for: Characterization and Expression Profiling of Camellia sinensis Cinnamate 4-hydroxylase Genes in Phenylpropanoid Pathways
Source: Genes (Basel). 2017 Aug 1;8(8):193. doi: 10.3390/genes8080193 (PMC5575657; doi:10.3390/genes8080193)

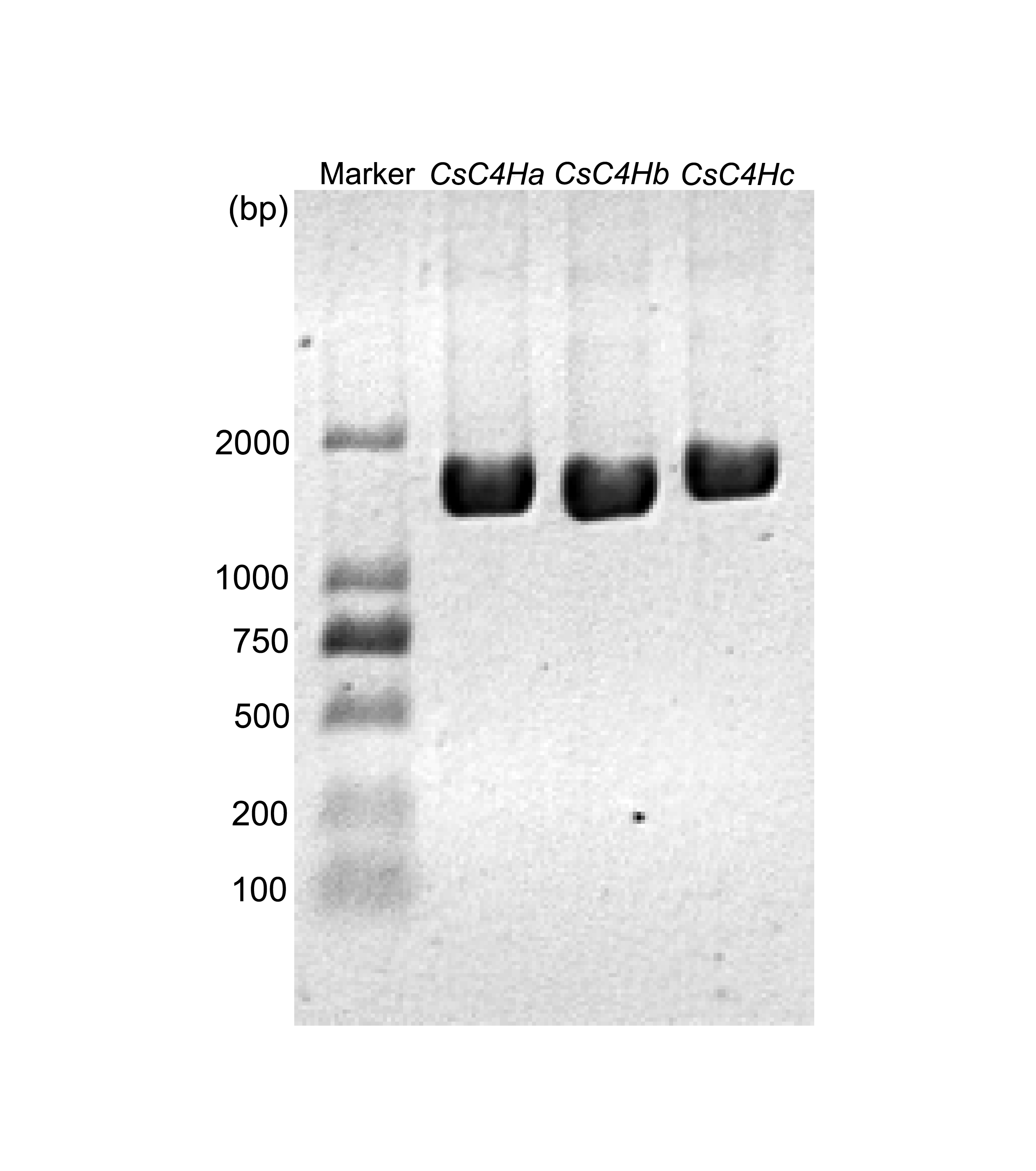

Supplement: Supplementary file 1 [file genes-08-00193-s001.tif]
